# HydroAir: An Air-Propelled Surface Vehicle for Autonomous Navigation and 3D Reconstruction in Shallow and Obstacle-Rich Aquatic Environments

**DOI:** 10.3390/s26103225

**Published:** 2026-05-20

**Authors:** Leonardo de Mello Honório, Vinícius Ferreira Vidal, Iago Zanuti Biundini, Rodolfo Almeida Machado, Felippe Fernandes, Murillo Ferreira dos Santos

**Affiliations:** 1 Faculty of Engineering, Federal University of Juiz de Fora (UFJF), Juiz de Fora 36036-900, Brazil; leonardo.honorio@ufjf.edu.br (L.d.M.H.); viniciusvidal2@gmail.com (V.F.V.); iago.biundini@gmail.com (I.Z.B.); rodolfo.mesalva@gmail.com (R.A.M.); 2 Axia Energy, Rio de Janeiro 20090-010, Brazil; felippefernandes@axia.com.br; 3 Master Degree Program in Automation and Systems (PPGAS), Department of Electroelectronics, Federal Center of Technological Education of Minas Gerais (CEFET-MG), Leopoldina 36700-001, Brazil

**Keywords:** air-propelled USV, overactuated vehicle, bathymetric mapping, 3D environmental reconstruction

## Abstract

This paper presents HydroAir, a novel air-propelled Unmanned Surface Vehicle (USV) specifically designed for operation in shallow waters and obstacle-rich aquatic environments such as lakes, reservoirs, and large dams. Unlike conventional aquatic robots, HydroAir employs an aerial propulsion system that enables it to overcome partially submerged obstacles, vegetation, and extremely shallow regions where traditional propeller-based platforms fail. The vehicle features a system with a very reliable internal architecture, providing high maneuverability and robustness in both manual and autonomous navigation modes. The primary objective of HydroAir is to serve as a mobile sensing platform for three-dimensional reconstruction of aquatic environments, particularly the underwater terrain. The onboard sensing suite enables bathymetric data acquisition, while a dedicated monitoring and control software integrates these data with aerial reconstructions obtained from Unmanned Aerial Vehicles (UAVs), allowing for the fusion of above-water and underwater spatial information into a unified 3D model. Experimental validations were conducted in large-scale, real-world environments, including tests in a hydroelectric dam operated by Santo Antônio Energia on the Madeira River in Brazil, demonstrating the platform’s operational feasibility, stability, and reconstruction capabilities. The results indicate that HydroAir is a promising solution for environmental monitoring, inspection, and mapping in challenging aquatic environments where conventional autonomous surface vehicles are limited.

## 1. Introduction

The monitoring and characterization of inland and coastal water bodies are critical for water-resource management, dam safety, environmental assessment, and infrastructure inspection. Large reservoirs, lakes, and hydropower dams often exhibit shallow zones, dense aquatic vegetation, debris, and partially submerged structures that hinder safe and repeatable access by conventional survey vessels. In such environments, producing accurate three-dimensional representations of underwater topography and associated structures is essential to support operation, maintenance, and risk analysis. However, this task remains technically challenging due to navigation constraints, collision risks, and the limitations of traditional propeller-based surface platforms [1,2]. At the same time, there is a growing demand for integrated observation systems that combine in situ sensing with advanced remote sensing and autonomous platforms, enabling continuous and multi-scale environmental monitoring across air, land, and water domains [3,4].

The Unmanned Surface Vehicles (USVs) have emerged as key enablers for automated bathymetry, inspection, and environmental data collection, offering persistent, cost-effective, and low-risk operations across a wide range of marine and inland water scenarios [1,2,5]. Recent surveys highlight substantial advances in USV guidance, navigation, and control, as well as in path planning, which have expanded their applicability to tasks such as environmental monitoring, surveillance, and marine science [1,6]. In parallel, UAVs have become a mature technology for high-resolution topographic mapping, environmental monitoring, and three-dimensional reconstruction of above-water structures, supported by photogrammetric and LiDAR-based workflows [7,8,9]. The fusion of UAV-derived topography with USV-based bathymetry enables seamless topo-bathymetric elevation models, providing continuous digital elevation representations across land–water interfaces and significantly improving spatial coverage and accuracy in river basins and aquatic systems [3,4]. However, most existing USV platforms still rely on water-borne propulsion systems, which are vulnerable to grounding, entanglement, and performance degradation in extremely shallow, obstacle-rich, or vegetated environments. As a result, critical near-shore and cluttered regions often remain poorly mapped [1,2].

These limitations motivate the development of novel surface platforms capable of robust operation in shallow, obstacle-dense environments while simultaneously serving as mobile sensing nodes within integrated UAV–USV mapping frameworks. This paper introduces HydroAir, an air-propelled Unmanned Surface Vehicle (USV) specifically designed to traverse very shallow waters, dense vegetation, and partially submerged obstacles, enabling access to regions that are typically unreachable by conventional surface vehicles. HydroAir features an overactuated and robust propulsion and control architecture, combined with an onboard bathymetric sensing suite and dedicated monitoring and control software. The proposed system supports the fusion of underwater terrain data with aerial reconstructions obtained from UAVs, producing a unified three-dimensional representation of the environment. This approach aligns with recent trends toward seamless topo-bathymetric modeling and integrated large-scale environmental monitoring [3,4,7]. The paper details the mechanical and systems design of the vehicle, the sensing and software architecture, and experimental validation conducted in large-scale real-world scenarios, including tests performed at the Santo Antônio hydropower reservoir in Brazil, demonstrating the platform’s operational feasibility, stability, and 3D reconstruction capabilities in challenging aquatic environments.

### 1.1. State of the Art and Related Works

Unmanned Marine Vehicles (UMVs), encompassing USVs, Unmanned Underwater Vehicles (UUVs), and Autonomous Underwater Vehicles (AUVs), have become central tools for maritime science, industrial operations, and defense applications, largely driven by advances in artificial intelligence, communication systems, and sensing technologies [2,10]. Recent surveys consistently report a strong shift toward higher levels of autonomy, cooperative multi-vehicle missions, and the integration of heterogeneous platforms operating across air, surface, and subsurface domains, particularly for large-scale and long-term environmental monitoring tasks [2,3,11].

Within this broader context, research on USVs has been predominantly focused on guidance, navigation, and control, with particular emphasis on path planning and collision avoidance in complex, dynamic, and partially structured environments [1,12,13,14,15]. Comprehensive surveys classify a wide spectrum of global and local path-planning approaches, including graph-based methods (e.g., A*, D*), sampling-based planners (e.g., RRT variants), artificial potential fields, velocity-obstacle formulations, fuzzy logic, and reinforcement learning. These works commonly evaluate performance using metrics such as path optimality, smoothness, convergence speed, computational cost, and compliance with maritime navigation rules (COLREGs) [12,13,14,15]. Scientometric analyses further indicate a steady growth in USV-related publications since the early 2000s, with an increasing proportion of studies validated through real-world experiments rather than purely simulation-based evaluations [13,15].

From an application standpoint, environmental and aquatic ecosystem monitoring has emerged as one of the fastest-growing domains for unmanned vehicles. A recent systematic review reports a twelvefold increase in the use of Unmanned Vehicle Systems (UVS) (including UAVs, USVs, and UUVs) for aquatic monitoring between 2013 and 2023 [11]. Approximately 70% of the surveyed studies rely on UAVs for large-area observation and surface mapping, while USVs and UUVs are primarily employed for in situ measurements of submerged habitats and water-quality parameters. Most applications focus on habitat mapping and animal behavior (exceeding 60%), whereas fewer than 10% explicitly address phenomena such as algal blooms or eutrophication. Moreover, tightly integrated UAV–USV missions remain relatively rare, largely due to persistent challenges in communication, coordination, and multi-modal data fusion [11].

At the system and deployment level, recent reviews of operative and market-ready USVs platforms highlight clear trends toward increased endurance, improved communication robustness, and modular payload architectures. At the same time, they identify a persistent gap between the sophistication of autonomy algorithms proposed in academic research and the comparatively conservative autonomy levels implemented in commercial systems [16]. Complementary surveys on integrated environmental monitoring frameworks emphasize that future solutions will increasingly depend on cooperative air–land–water sensing networks, combining UAVs, USVs, UUVs/AUVs, wireless sensor networks, and advanced signal processing to enable continuous, multi-scale observations of complex environments [3].

Despite these advances, there remains limited coverage of USVs specifically optimized for operation in very shallow, obstacle-rich, and vegetated reservoirs, and explicitly conceived as mobile bathymetric sensing nodes within tightly coupled UAV–USV three-dimensional reconstruction pipelines. Addressing this gap is the primary motivation behind the HydroAir concept proposed in this work.

Building upon the previously discussed state of the art, several representative and influential works help to contextualize the HydroAir concept within the broader research landscape of unmanned marine vehicles, autonomous navigation, and integrated environmental monitoring. These works collectively define the technological foundations, algorithmic advances, and system-level limitations that motivate the development of novel surface platforms targeting challenging aquatic environments.

A comprehensive perspective on the evolution of unmanned marine vehicles is provided by Bae and Hong [2], which surveys both USVs and UUVs with a strong emphasis on intelligence, autonomy, and cooperative operation. That work synthesizes developments ranging from early experimental platforms to recent Artificial Intelligence (AI)-enabled systems and organizes the field around sensing technologies, autonomous decision-making, and multi-vehicle cooperation. Quantitative trends reported therein indicate a substantial growth in research output, while also showing that most operational demonstrations still involve relatively small numbers of cooperating vehicles and task-specific scenarios. Although Bae and Hong [2] does not focus on a particular vehicle architecture, its conclusions underscore the importance of robust sensing, communication, and autonomy, which are partially addressed in HydroAir through modular payloads and dedicated monitoring and control software. In contrast to the system-level and cooperative focus of that review, HydroAir focuses on a single-vehicle solution optimized for shallow, cluttered waters and on tight integration with UAV-based mapping rather than large-scale swarm coordination.

Foundational aspects of USV development and guidance, navigation, and control are extensively reviewed in Liu et al. [1]. That work classifies USVs by application domain and control strategy, covering Line of Sight (LoS) guidance, model-based control, and adaptive and intelligent approaches. Case studies compiled in the review demonstrate that conventional USVs can achieve meter-level tracking accuracy and multi-hour endurance in open-water conditions, while also highlighting persistent challenges related to robustness under environmental disturbances and scalability to larger platforms. HydroAir builds on these established GNC principles but departs from the dominant design paradigm by employing air propulsion rather than water-borne propellers. This shift alters the actuation characteristics and disturbance sensitivity, potentially reducing the risk of grounding and entanglement in very shallow or debris-filled waters, while introducing new challenges related to wind effects and energy efficiency that are less prominent in the platforms surveyed in Liu et al. [1].

High-level navigation, route planning, and collision avoidance for autonomous surface vessels are further examined in surveys such as Vagale et al. [15], which analyze algorithmic approaches in relation to autonomy levels and regulatory constraints, including COLREGs. That review reports performance gains in simulated environments, including reduced path length and reduced collision risk. Still, it emphasizes that only a fraction of the proposed methods have been validated in real maritime conditions. From HydroAir’s perspective, these algorithmic contributions form a mature toolbox that can be adapted for shallow-water missions. However, many of the reviewed methods implicitly assume open or coastal waters and relatively unconstrained maneuverability, conditions that differ markedly from densely vegetated reservoirs and near-shore environments. Consequently, while HydroAir can leverage existing path-planning and collision-avoidance strategies, practical deployment in its target scenarios may require adaptations to account for tight clearances, non-holonomic constraints, and the dynamics of air-propelled actuation.

A more focused analysis of route-planning algorithms for USVs over the period 2000–2023 is presented in Hashali et al. [13], combining scientometric methods with a comparative assessment of global and local planners validated in simulation and field trials. That study highlights hybrid and AI-based approaches as particularly promising for real-world deployment, while noting that field validation remains limited. Relative to this body of work, HydroAir does not introduce a novel planning algorithm, but instead provides a new vehicle class and operational niche in which these algorithms can be applied. The distinctive dynamics and motion constraints of HydroAir imply that planners developed for conventional propeller-driven USVs may require re-tuning or redesign, yet the platform’s ability to physically access shallow and cluttered regions offers a complementary contribution to the algorithm-centric advances summarized in Hashali et al. [13].

The gap between academic research and deployable systems is examined in de Andrade et al. [16], which surveys market-ready USVs and analyzes trends in propulsion, endurance, autonomy, and application domains. That review shows that many commercial platforms still rely on relatively simple autonomy modes, despite extensive academic progress in AI-based navigation and control. It also reveals that most operative USVs are optimized for open-sea or deep-water coastal missions and employ conventional water-borne propulsion. In this context, HydroAir represents a departure from prevailing market trends by explicitly targeting very shallow, vegetated, and obstacle-rich reservoirs using air propulsion. While this specialization limits its suitability for long-endurance offshore missions, it addresses a niche that remains underserved by existing commercial platforms.

Finally, the broader application domain of aquatic ecosystem monitoring is reviewed in Liu et al. [11], which reports a rapid growth in the use of UVSs for multi-scale environmental observation and highlights the dominance of UAVs in large-area mapping tasks. The review identifies integrated UAV–USV missions as an under-represented but promising direction, constrained by challenges in communication, coordination, and data fusion. Complementary perspectives on integrated monitoring systems are provided in [3], which emphasize cooperation among aerial, terrestrial, and aquatic sensing platforms to achieve continuous and multi-scale observations. HydroAir directly addresses the integration gap identified in these works by acting as a shallow-water bathymetric node explicitly designed to complement UAV-based topographic mapping. While the platform does not yet match the breadth of ecological applications covered across the wider UVSs literature, it provides a concrete hardware–software solution for seamless three-dimensional reconstruction of complex aquatic environments, aligning with the long-term vision of integrated air–water environmental monitoring systems.

In addition to navigation and coverage constraints, several studies highlight that disturbances at the air–water interface and turbulence of the free surface can significantly degrade the performance of aquatic sensing systems. Experimental analyses show that increased surface roughness and turbulent fluctuations introduce noise, signal loss, and bias in ultrasonic, optical, and laser-based measurements, directly affecting the reliability of bathymetric and environmental data [17,18]. These effects are particularly relevant for mobile platforms, where propulsion-induced waves, wake turbulence, and surface agitation may dominate the measurement error budget, even when advanced sensors are employed. Consequently, beyond algorithmic accuracy and sensor resolution, the hydrodynamic interaction between the vehicle and the free surface becomes a critical but often overlooked design factor in shallow-water monitoring missions.

Within this context, the HydroAir platform offers a practical advantage by decoupling propulsion from direct water interaction, thereby minimizing propulsor-induced turbulence in the water column and at the free surface. By relying on air propulsion, HydroAir reduces wake generation, sediment resuspension, and surface agitation in very shallow and vegetated environments, creating more favorable conditions for stable bathymetric sensing and data fusion with aerial observations. This characteristic directly addresses the sensor-interference mechanisms discussed in the literature and complements existing algorithmic and sensing advances with a vehicle-level solution. As a result, HydroAir exemplifies how appropriate platform design can enhance measurement quality and operational reliability in challenging aquatic environments, reinforcing its role as a practical and effective component of integrated UAV–USV three-dimensional reconstruction and environmental monitoring systems.

### 1.2. Objectives and Main Contributions

The main objective of this work is to develop, validate, and experimentally demonstrate a novel USV capable of reliable operation in shallow, obstacle-rich, and vegetated aquatic environments, while supporting accurate three-dimensional reconstruction of underwater and near-shore regions. To this end, the proposed platform, named HydroAir, is conceived as an air-propelled surface vehicle explicitly designed to minimize hydrodynamic disturbance at the air–water interface and to act as a mobile bathymetric sensing node within integrated aerial–aquatic mapping frameworks.

More specifically, this work aims to (i) enable safe and repeatable navigation in environments that are typically inaccessible or risky for conventional propeller-driven USVs, such as very shallow reservoirs, debris-filled areas, and regions with dense aquatic vegetation; (ii) reduce propulsion-induced turbulence, wake generation, and sediment resuspension in order to improve the quality and stability of onboard sensor measurements; and (iii) support the fusion of underwater terrain data with aerial reconstructions obtained from UAVs, enabling seamless topo-bathymetric three-dimensional models of complex aquatic environments.

The main contributions of this paper can be summarized as follows:

- The design of a novel air-propelled USV architecture specifically optimized for shallow-water and obstacle-rich environments, providing enhanced maneuverability and robustness compared with conventional water-propelled platforms;
- A vehicle-level approach to reducing sensor interference by minimizing hydrodynamic disturbance at the free surface, addressing an often overlooked source of measurement degradation in shallow-water bathymetric and environmental sensing;
- Experimental validation of the proposed platform in large-scale, real-world reservoir environments, demonstrating its operational feasibility, navigation stability, and suitability for three-dimensional reconstruction in challenging aquatic scenarios.

Together, these contributions position HydroAir as a practical and effective solution for environmental monitoring, inspection, and mapping in shallow, cluttered aquatic environments, complementing existing advances in autonomous navigation and sensing with a platform design explicitly tailored to the physical constraints of these environments.

### 1.3. Paper Organization

The remainder of this paper is organized as follows. Section 2 presents the development of the HydroAir prototype, including the mechanical structure, propulsion configuration, and the main onboard electronic components used in the experimental platform; Section 4 introduces the dynamic modeling of the surface vehicle, describing the kinematic and dynamic equations adopted to represent the motion of the platform; Section 5 details the control strategy employed for the vehicle, including the hierarchical control structure and the control allocation approach used to distribute the commanded forces among the available actuators; Section 6 presents the experimental results obtained with the HydroAir prototype in real aquatic environments, demonstrating the operational capabilities of the system and illustrating practical applications of the platform; finally, Section 7 summarizes the main contributions of this work and discusses possible directions for future research, also with some future works.

## 2. HydroAir Architecture and Functionalities

This section presents the development of the HydroAir prototype designed to experimentally validate the proposed modeling, control, and allocation framework under real operating conditions. The platform was conceived to provide a robust, modular, and reconfigurable structure capable of supporting diverse actuation strategies while maintaining mechanical stability and ease of integration between the hardware and the control architecture.

**Figure 1:**
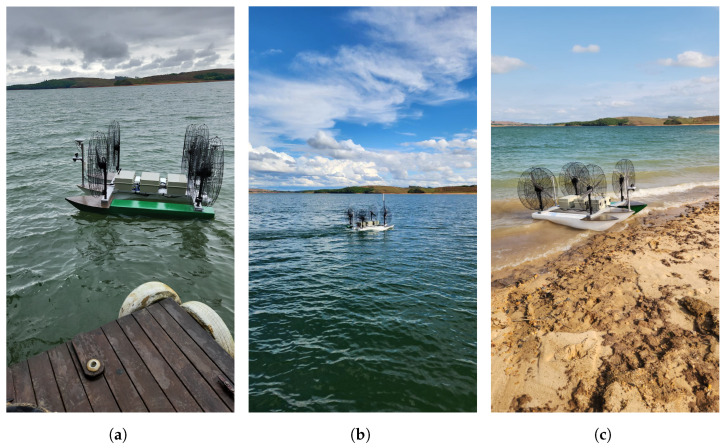
Experimental validation campaign in a lake environment demonstrating the real-world implementation of the proposed control architecture. (**a**) Experimental configuration prior to deployment. (**b**) Autonomous navigation during lake trials. (**c**) Post-trial recovery at shoreline.

Figure 1 illustrates the developed HydroAir platform during experimental validation in a controlled real-world environment. It is important to note that, during the early stages of system maturity, the experimental tests were conducted outside the Santo Antônio deployment site, before progressing to in-situ validation under real operational conditions.

The vehicle adopts a trimaran configuration, consisting of two or three parallel hulls (depending the sonar configuration) mounted on a central structural frame, with a total length of 2.25 m, a width of 2.3 m, and an overall height of 1.4 m. The total mass of the platform is approximately 192.9 kg, including the propulsion system, onboard electronics, and power supply. The triple-hull architecture was selected to improve lateral stability, enhance buoyancy distribution, and provide a wide base for the symmetric placement of propulsion units. This configuration increases roll stability and reduces undesired oscillations during maneuvering, particularly at low speeds.

The structural frame is manufactured from high-performance composite materials, including carbon fiber, aramid fiber, and fiberglass, selected for their favorable stiffness-to-weight ratios and corrosion resistance in freshwater environments. Each hull provides a buoyant force of approximately 687 N, corresponding to a displaced freshwater volume of 70 L. Considering the three-hull configuration, the total buoyant force is approximately 2060 N, equivalent to a maximum supported mass of approximately 210 kg under static equilibrium conditions. The center of mass is approximately 0.5 m above the waterline and 0.1 m from the geometric center along the longitudinal axis, which improves dynamic balance.

The propulsion system consists of six aerial thrusters symmetrically mounted along the vehicle’s longitudinal axis, with the two rear thrusters oriented 180° relative to the other four. Each thruster delivers approximately 200 N of thrust and operates over a 48 V to 50.4 V voltage range. This symmetric configuration enables the generation of control forces in the surge direction and control moments around the yaw axis through differential thrust.

The adopted actuation arrangement results in a reliable planar motion control, since multiple actuator combinations can produce equivalent generalized forces. This characteristic motivates the use of a control allocation strategy, which optimally distributes the control effort among the available thrusters. The geometric placement of the actuators defines the control effectiveness matrix, directly linking actuator inputs to the generalized force vector composed of surge and sway forces and the yaw moment. The design intentionally preserves this redundancy in order to improve robustness against actuator saturation and potential faults.

Electrical power is supplied by two battery packs, each with a nominal capacity of 64 Ah and a nominal voltage of 22.2 V, providing an estimated operational autonomy of approximately 180 min under nominal operating conditions. Power distribution is regulated through six Electronic Speed Controllers (ESCs), each capable of handling currents up to 120 A. The propulsion subsystem was dimensioned to ensure that the maximum available thrust exceeds the force required for steady-state navigation at the desired cruise speed of 1 m/s.

The mechanical and electronic subsystems were designed to directly support the hierarchical control architecture presented in the previous section. In particular, the propulsion layout and actuator symmetry were defined to facilitate the implementation of allocation-based control strategies, enabling efficient mapping between high-level control objectives and low-level actuator commands.

The onboard computational architecture comprises an embedded processing unit operating at 2.4 GHz with 16 GB of RAM, responsible for the real-time execution of control algorithms, sensor fusion, and actuator command generation. State estimation is obtained by integrating a GNSS receiver with approximately 2 m accuracy and an Inertial Measurement Unit (IMU) that provides tri-axial acceleration and angular velocity measurements sampled at 1 kHz. The control architecture operates in a hierarchical structure, with a low-level control loop running at 400 Hz and a high-level guidance and navigation layer running at 80 Hz, ensuring adequate responsiveness for maneuvering tasks.

Communication between sensors, the processing unit, and actuators is implemented via a combination of SPI, I^2^C, UART, and CAN buses, while external telemetry and command exchange use the MAVLink protocol. The overall system architecture was designed to minimize computational and communication delays, resulting in an estimated end-to-end control latency of approximately 10 ms.

**Figure 2:**
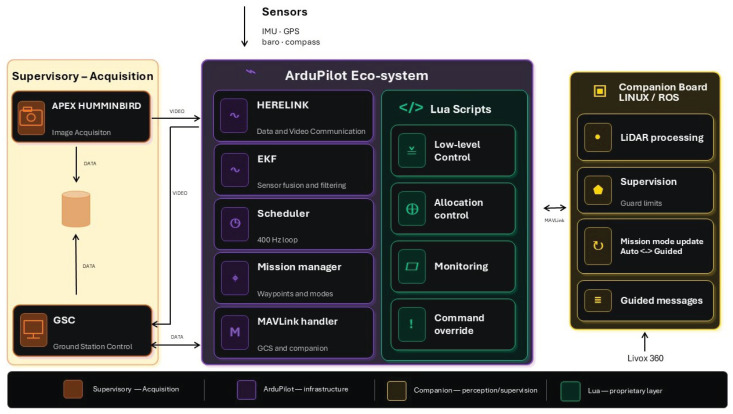
System architecture.

Figure 2 presents a high level view of the proposed system architecture. It is organized into four main functional layers: the supervisory-acquisition layer, the ArduPilot eco-system, the companion board, and the proprietary Lua-based control layer embedded within ArduPilot. This organization separates image acquisition, flight-control infrastructure, high-level perception and supervision, and low-level actuation logic, while maintaining communication through MAVLink and dedicated video/data links.

The supervisory-acquisition layer is responsible for operator interaction, mission monitoring, and image acquisition. In the implemented configuration, this layer includes the Ground Station Control (GSC) and the Humminbird APEX unit. The GSC acts as the human–machine interface for mission supervision, visualization, and operational monitoring, while the Humminbird APEX operates as an image-acquisition subsystem, providing visual information that can be monitored by the operator and incorporated into the supervision workflow.

This layer is sensor-dependent and may require different hardware and software arrangements depending on the payload adopted. For example, a Teledyne Marine MB2 sonar would require a different acquisition architecture, including an onboard computer running Windows to configure, control, and acquire data from the sensor. In the present configuration, communication between the GSC and the acquisition subsystem is implemented through dedicated video and data channels, allowing the operator to observe the environment while maintaining mission-level awareness.

The core navigation and vehicle-control infrastructure is implemented within the ArduPilot eco-system. The Herelink module provides data and video communication, acting as an interface between the field system and the ground station. The Extended Kalman Filter (EKF) performs sensor fusion and filtering using onboard measurements from the IMU, GPS, barometer, and compass. These estimates provide the navigation state required by the control and mission-management layers. The scheduler executes the real-time control loop at 400 Hz, ensuring deterministic execution of the embedded control routines. The mission manager handles waypoints and navigation modes, while the MAVLink handler manages communication with the ground station and the companion board.

A proprietary Lua-based layer runs inside the ArduPilot environment and implements vessel-specific control functions. This layer includes the low-level control logic, the control-allocation module, monitoring routines, and command override mechanisms. The low-level controller computes the required actuation commands for the vessel, while the allocation controller maps the demanded forces and moments into the available propulsion configuration. The monitoring block evaluates operational conditions and internal control states, and the command override block allows the Lua layer to impose priority commands when required for safety or mission execution. By placing this layer inside ArduPilot, the system preserves the robustness and maturity of the autopilot infrastructure while allowing vessel-specific customization.

The companion board runs a Linux/ROS environment and is responsible for perception and high-level supervision tasks. It processes data from the Livox 360 LiDAR and executes supervision routines associated with guard limits, mission-mode management, and guided-command generation. The LiDAR-processing module provides environmental perception for obstacle detection and situational awareness. The supervision module evaluates safety constraints and operational limits. When required, the companion board updates the mission mode, for instance by switching from Auto to Guided, and generates guided messages to modify the vehicle behavior in real time. Communication between the companion board and ArduPilot is performed through MAVLink.

This architecture allows for the autonomous vessel to combine consolidated autopilot functions with external perception, customized low-level control, and mission-oriented data acquisition. ArduPilot provides the real-time navigation backbone, sensor fusion, mission execution, and communication infrastructure. The Lua scripts implement the proprietary control logic required by the specific propulsion arrangement of the vessel, while the companion board adds computational capacity for LiDAR-based perception and supervisory decision-making. Finally, the supervisory-acquisition layer provides the operator with mission monitoring, image acquisition, and ground-station interaction.

**Figure 3:**
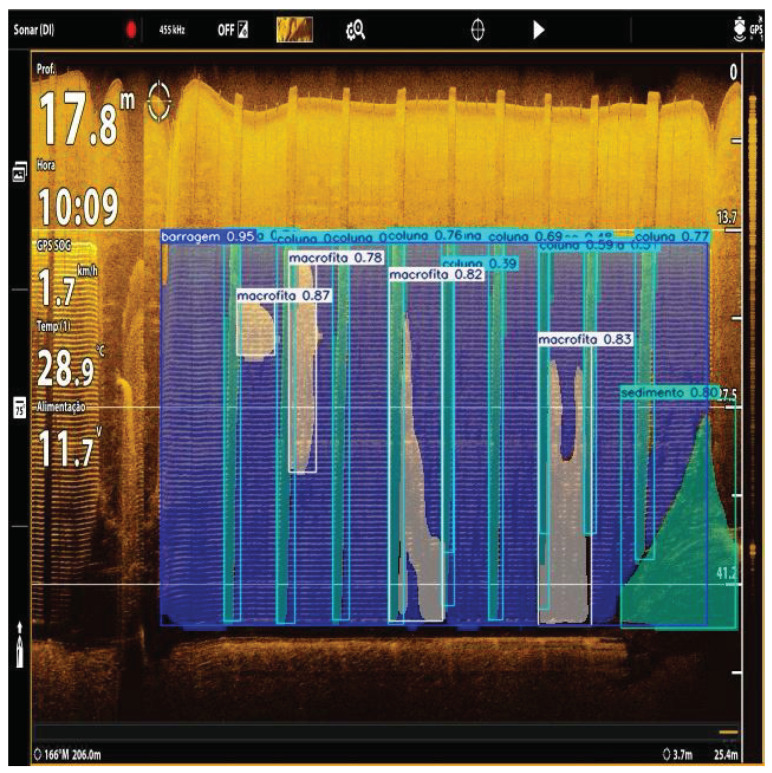
Post-processed sonar imagery of a hydropower trash rack.

Although the main objective of this paper is not to present the full results of the data-acquisition campaign, but rather to describe the vessel topology and its control architecture, the sonar-acquisition capability illustrates the operational relevance of the proposed platform. By safely navigating close to the dam and to the intake structures, the vessel can acquire high-resolution sonar imagery of the hydropower plant trash racks. Using software tools developed by the team, these images can be processed to estimate the volume, area, and morphology of debris accumulated on the racks, including macrophytes, sediment deposits, and other obstructing material as shown in Figure 3. Such deposits increase hydraulic head losses, reduce generation efficiency, and may lead to significant operational and maintenance costs. Therefore, the proposed vessel provides a safe and repeatable means of acquiring field data to support inspection routines, quantify obstruction levels, and optimize maintenance planning at the hydropower plant.

This modular organization improves maintainability, enables incremental development, and allows safety-critical functions to be distributed between embedded control, external supervision, and operator-level monitoring, while also supporting payload-specific acquisition workflows for hydropower inspection and maintenance optimization.

## 3. Operational Environment and Field Conditions

### 3.1. The Environment

The Santo Antônio Hydroelectric Power Plant (UHE Santo Antônio) is located at the Santo Antônio Falls on the Madeira River, approximately 6 km upstream from the city of Porto Velho, capital of the state of Rondônia, at the geographic coordinates 8°48′04″ S and 63°57′08″ W. It is a large-scale hydropower development, with an installed capacity of 3568 MW and a long-term mean flow (MLT) of 18,500 m^3^/s, ranking among the largest hydroelectric plants currently in operation in Brazil.

The layout of the structures and generating units follows the river’s transverse profile. It consists of 24 generating units (GUs) located on the left bank, 18 in the central structure within the riverbed, and 8 on the right bank, totaling 50 GUs, all connected to the National Interconnected System. The hydraulic arrangement also includes the Main Spillway, the Auxiliary Spillway, the Fish Passage System (FPS), the Log Spillway, and the Log Boom, the latter being essential for controlling the substantial volume of woody debris and floating organic material transported by the river.

The Madeira River is the largest right-bank tributary of the Amazon River and the 17th longest river in the world, with approximately 1450 km in length and accounting for about 20% of the total area of the Amazon Basin. Its drainage basin covers approximately 1,000,000 km^2^, distributed across Bolivia (74%), Brazil (14%), and Peru (12%), receiving contributions from its main tributaries along the Brazil–Bolivia border before reaching the hydropower plant axis.

From a hydro-sedimentological perspective, the Madeira River is one of the most sediment-rich large rivers in the world. Published estimates indicate an annual suspended sediment load in the order of 430 to 600 million tonnes per year, with some earlier estimates reaching approximately 715 million tonnes per year. This places the Madeira among the major global sediment-transport rivers and makes it the dominant sediment contributor within the Amazon system, accounting for roughly 45–50% of the Amazon River sediment load despite representing a much smaller fraction of the basin discharge.

In addition to its high sediment load, the Madeira River also transports a significant amount of floating organic material, including trunks, branches, aquatic vegetation, and macrophyte mats, particularly during high-flow periods. Although public sources do not provide a single consolidated annual tonnage for macrophyte biomass at the Santo Antônio site, the presence of dedicated structures such as the Log Boom and the Log Spillway demonstrates the operational relevance of this floating material. In practical terms, the Madeira can be classified as a river with very high sediment load and high floating-debris and aquatic-vegetation transport, creating persistent challenges for hydropower operation, intake protection, reservoir management, inspection routines, and environmental monitoring.

### 3.2. Field Navigation Conditions

**Figure 4:**
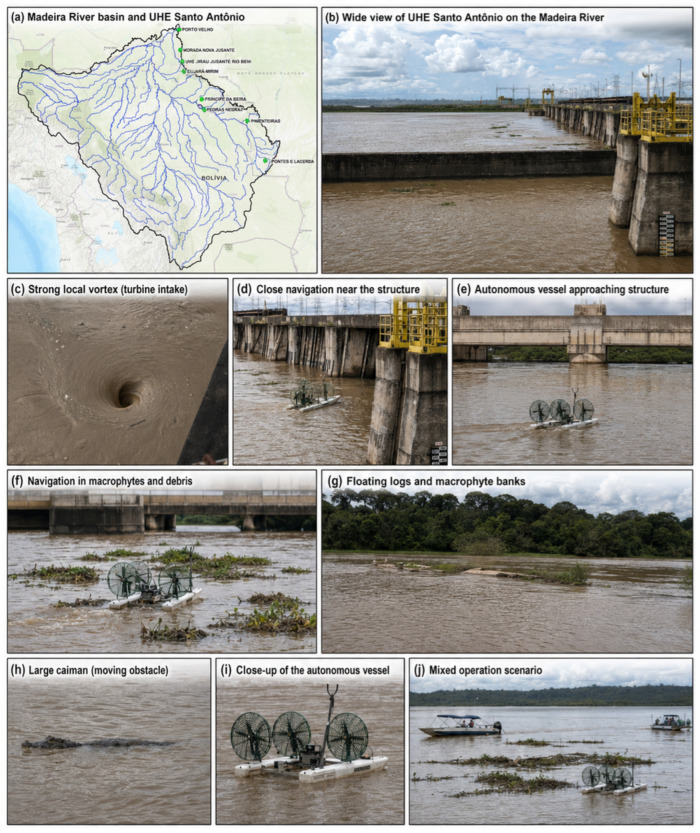
Representative field conditions during autonomous vessel trials at the Santo Antônio Hydroelectric Power Plant on the Madeira River, including hydropower structures, strong local vortices, floating debris, macrophyte banks, close-proximity dam navigation, and mobile biological obstacles.

Figure 4 summarizes the main environmental and operational conditions encountered during the autonomous vessel initial trials at the Santo Antônio Hydroelectric Power Plant, on the Madeira River. This deployment site differs substantially from conventional experimental areas commonly used for autonomous surface vessel validation, such as calm lakes, protected canals, urban waterways, or coastal test fields. The vessel was tested in a real hydropower operating environment, directly affected by dam structures, turbine water intake, sediment transport, floating debris, aquatic vegetation, and local wildlife.

One of the most critical aspects of this environment is the proximity to the dam and to the hydraulic structures of the power plant. In these regions, the flow field is highly nonuniform and may change abruptly over short distances. Both the magnitude and direction of the current can vary significantly along the navigation axis, especially near turbine intake zones and structural discontinuities. These flow variations impose strong disturbances on the vessel, affecting heading control, trajectory tracking, station keeping, and obstacle avoidance. Unlike navigation in open and slowly varying water bodies, the vessel must continuously compensate for localized hydrodynamic effects generated by the plant operation.

The operating conditions become even more severe when turbine water intake increases. Under these circumstances, intense local vortices may appear near the structures, producing strong rotational flow patterns and localized suction effects. These vortices represent a relevant operational hazard because they can rapidly modify the vessel trajectory and create regions where manual navigation would be unsafe or impractical. In several sectors close to the dam, the autonomous vessel navigates in areas where crewed boats are not considered safe due to the combined risk of strong currents, abrupt flow direction changes, structural proximity, and the possibility of collision with submerged or semi-submerged obstacles.

Another important characteristic of the Madeira River is the large amount of suspended sediment and floating organic material transported by the flow. The water is highly turbid, which limits visual perception and makes it difficult to identify submerged hazards using conventional observation. In addition, the river carries trunks, branches, macrophyte mats, and other floating debris. The autonomous vessel was therefore required to operate not only in open water, but also in cluttered regions containing floating vegetation and woody material. The vehicle can navigate over small floating trunks and macrophyte banks when they do not represent a critical risk to the hull, propulsion system, or onboard sensors. However, larger obstacles are treated as hazards and must be detected and avoided to preserve the integrity of the sensing payload and navigation system.

The presence of macrophyte banks and floating debris also creates a dynamic and partially unstructured navigation scenario. These obstacles are not fixed elements of the environment: they move with the current, accumulate near hydraulic structures, disperse according to local turbulence, and may suddenly enter the planned trajectory. As a result, the navigation problem involves both static constraints, such as dam walls and concrete structures, and mobile or deformable obstacles, such as vegetation mats and floating logs. This combination requires a robust low-level control system, fast supervisory logic, and conservative safety rules for deciding when the vessel should cross, avoid, or replan around an obstacle.

In addition to hydrodynamic and debris-related hazards, the deployment area includes biological moving obstacles, such as large caimans. These animals represent an additional source of uncertainty because their motion is not predictable and they may appear close to the planned path. From the perspective of autonomous navigation, they must be treated as dynamic obstacles whose avoidance is necessary both for operational safety and for environmental protection. This reinforces the need for a perception and supervision layer capable of reacting to unexpected events in real time.

These conditions make the Santo Antônio deployment site one of the most demanding environments evaluated in this work. The vessel operates in highly sediment-laden waters, over small floating debris and macrophyte patches, close to large hydropower structures, and under strongly varying currents induced by dam operation and turbine intake. In practical terms, the system is required to operate in regions that are often unsuitable for crewed vessels, while preserving onboard sensors, avoiding larger obstacles, and maintaining controllability under severe disturbances.

Although the literature presents excellent autonomous surface vessel solutions in different scenarios, including prototype navigation and control [19], autonomous operation in urban waterways [20], and long-endurance wind- and solar-powered platforms [21], the operating conditions reported in those works are substantially different from the combination observed at Santo Antônio. To the best of our knowledge, no prior work reports autonomous surface navigation under the specific combination of high sediment load, macrophyte banks, floating woody debris, close-proximity navigation near hydropower structures, turbine-induced vortices, strong spatial current gradients, and large mobile biological obstacles.

## 4. HydroAir Kinematics and Dynamics Modeling

The dynamic representation of the proposed USV follows the classical modeling assumptions commonly adopted for surface vessels, in which the motion is predominantly described by 3 Degree of Freedom (DoF). These correspond to the translational movements along the longitudinal and lateral axes, known as Surge and Sway, respectively, and the rotational motion about the vertical axis, referred to as Yaw. The remaining DoFs are neglected, as their influence on the overall dynamics is comparatively small for the operational conditions considered in this work [22].

**Figure 5:**
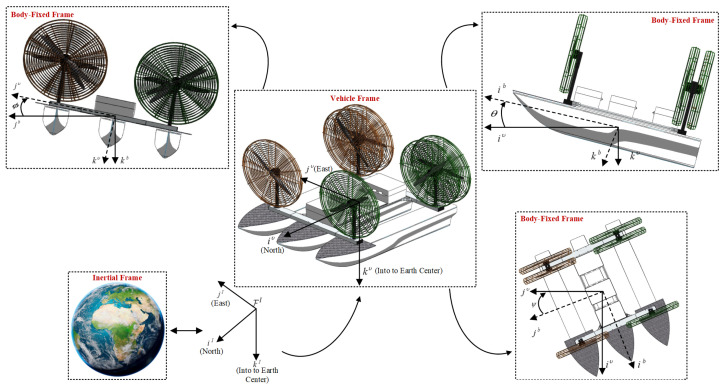
Reference frames and variables adopted for HydroAir.

Before starting its kinematics and dynamics modeling, it is necessary to clarify the reference frames adopted. Then, Figure 5 illustrates the coordinate systems and associated variables considered in this study.

In this representation, the orientation angles roll, pitch, and yaw (ϕ,θ,ψ) are defined with respect to the inertial frame $F^{I}$, whose axes are denoted by $\left({\hat{i}}^{I},{\hat{j}}^{I},{\hat{k}}^{I}\right)$. The vehicle frame $F^{\upsilon }$ is obtained through a translational transformation of $F^{I}$, while the body-fixed frame $F^{b}$, with axes $\left({\hat{i}}^{b},{\hat{j}}^{b},{\hat{k}}^{b}\right)$, is attached to the vessel and moves consistently with its rigid-body motion.

According to standard marine vehicle notation, two state vectors are defined. The first one, $\eta ={\left[x,y,\psi \right]}^{T}$, represents the vehicle position and orientation expressed in the inertial reference frame $F^{I}$, where *x* and *y* denote planar coordinates and ψ corresponds to the heading angle. The second vector, $\nu ={\left[u,v,r\right]}^{T}$, contains the linear and angular velocities expressed in the body-fixed frame $F^{b}$, where *u* and *v* are the surge and sway velocities, and *r* is the yaw rate [22].

Under these assumptions, the kinematic and dynamic behavior of the USV, disregarding external disturbances, can be described by the following nonlinear equations [23]:

(1) $$\begin{array}{ccc} M\dot{\nu }+C\left(\nu \right)\nu +D\left(\nu \right)\nu & = & \tau , \end{array}$$

(2) $$\begin{array}{ccc} \dot{\eta } & = & J(\psi )\nu , \end{array}$$

where $M\in R^{3\times 3}$ denotes the inertia matrix including added mass effects, $C\left(\nu \right)\in R^{3\times 3}$ represents the Coriolis and centripetal terms, $D\left(\nu \right)\in R^{3\times 3}$ accounts for hydrodynamic damping, and $\tau \in R^{3\times 1}$ is the vector of generalized forces and moments generated by the propulsion system. The transformation between the body-fixed and inertial frames is performed by the Jacobian matrix J(ψ).

Considering the coupling effects among the three DoFs, the inertia, Coriolis, and kinematic matrices, the modeling assumes the following forms [23]:

(3) $$\begin{array}{ccc} M & = & \left[\begin{array}{ccc} m-X_{\dot{u}} & -X_{\dot{v}} & -X_{\dot{r}}\\ -Y_{\dot{u}} & -Y_{\dot{v}} & mx_{g}-Y_{\dot{r}}\\ -N_{\dot{u}} & mx_{g}-N_{\dot{v}} & I_{z}-N_{\dot{r}} \end{array}\right], \end{array}$$

(4) $$\begin{array}{ccc} C(\nu ) & = & \left[\begin{array}{ccc} 0 & 0 & -\beta_{2}\\ 0 & 0 & \beta_{1}\\ \beta_{2} & -\beta_{1} & 0 \end{array}\right], \end{array}$$

(5) $$\begin{array}{ccc} J(\psi ) & = & \left[\begin{array}{ccc} \cos(\psi ) & -\sin(\psi ) & 0\\ \sin(\psi ) & \cos(\psi ) & 0\\ 0 & 0 & 1 \end{array}\right], \end{array}$$

where *m* represents the total mass of the vessel, $I_{z}$ is the moment of inertia about the vertical axis, and $x_{g}$ denotes the longitudinal position of the center of gravity. The coefficients $X_{\dot{\left(\cdot \right)}},Y_{\dot{\left(\cdot \right)}},N_{\dot{\left(\cdot \right)}}$ correspond to the added mass hydrodynamic derivatives. The auxiliary terms $\beta_{1}$ and $\beta_{2}$ group velocity-dependent contributions arising from rigid-body and added-mass effects.

Hydrodynamic damping is modeled as the superposition of linear and nonlinear components, with the latter associated with quadratic velocity terms. Thus, the damping matrix D(ν) is written as follows [23]:

(6) $$\begin{array}{cc} D\left(\nu \right)= & \left[\begin{array}{ccc} -X_{u} & -X_{v} & -X_{r}\\ -Y_{u} & -Y_{v} & -Y_{r}\\ -N_{u} & -N_{v} & -N_{r} \end{array}\right]+\left[\begin{array}{ccc} -X_{|u|u}\left|u\right| & -X_{|u|v}\left|u\right| & -X_{|u|r}\left|u\right|\\ -Y_{|v|u}\left|v\right| & -Y_{|v|v}\left|v\right| & -Y_{|v|r}\left|v\right|\\ -N_{|r|u}\left|r\right| & -N_{|r|v}\left|r\right| & -N_{|r|r}\left|r\right| \end{array}\right], \end{array}$$

where the first matrix contains the linear damping coefficients, while the second matrix represents quadratic damping effects.

The identification of the vessel parameters was carried out in two complementary stages. Initially, the inertial properties were obtained through direct measurements and supported by Computer-Aided Design (CAD) models. Subsequently, the hydrodynamic parameters associated with the matrices M, C(ν), and D(ν) were estimated using the rSOESGOPE method proposed in dos Santos Neto et al. [24] and Dos Santos et al. [25], which emphasizes robustness in parametric estimation. The resulting inertial and hydrodynamic parameters are summarized in Table 1 and Table 2:

It is worth noting that this model serves as a reliable foundation, while its parameters are adjusted to accurately represent the HydroAir prototype and its specific propulsion and operational characteristics.

## 5. Control Structure

This section presents the control architecture adopted for HydroAir for planar surface navigation, considering surge (u), sway (v), and yaw (ψ) dynamics. In the adopted implementation, the guidance layer generates desired linear and angular velocity references, while the low-level controllers regulate the corresponding surge and yaw dynamics.

The overall control structure is divided into two hierarchical levels: a high-level guidance layer, which converts waypoint and path-following objectives into desired linear and angular velocity commands; and a low-level control layer, which tracks these commands and generates the corresponding virtual control actions for the vessel.

**Figure 6:**
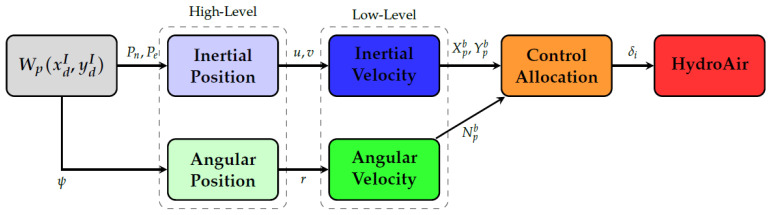
Hierarchical control architecture including position loops, angular loops, and the control allocation layer, where $P_{n}$ and $P_{e}$ are the North and East desired positions in the $F^{I}$, $\delta_{i}$ is the Pulse Width Modulation (PWM) of each propulsion motor, $X_{p}^{b}$ represents the commanded surge thrust (N), $Y_{p}^{b}$ the commanded sway (lateral) force (N), and $N_{p}^{b}$ the commanded yaw moment contribution (Nm), both in $F^{b}$.

Each controlled variable is implemented using a two-layer cascade structure composed of: (i) an external guidance controller; (ii) an internal Proportional, Integral and Derivative (PID) velocity controller. Figure 6 illustrates this.

Let the inertial position error be defined as

(7) $$e_{\eta }=\left[\begin{array}{c} x_{d}^{I}-x^{I}\\ y_{d}^{I}-y^{I} \end{array}\right].$$

The external proportional controller generates desired inertial velocities:

(8) $$\nu_{d}^{I}=K_{p}^{\eta }e_{\eta },$$

where $K_{p}^{\eta }=\mathrm{diag}\left(k_{p}^{x},k_{p}^{y}\right)$.

The desired inertial velocities are then transformed into body-fixed references using

(9) $$\nu_{d}=R^{T}\left(\psi \right)\nu_{d}^{I}.$$

where R(ψ) denotes the planar rotation matrix associated with the vessel yaw angle ψ.

The transpose $R^{T}\left(\psi \right)$ therefore maps vectors from $F^{I}$ reference frame to $F^{b}$. This transformation is consistent with the standard kinematic formulation adopted for marine vehicles, where the relationship between inertial and body velocities is defined through the Jacobian matrix J(η) shown in Equation (2). In the planar case considered for the HydroAir vessel, this matrix reduces to a block structure in which the rotation matrix R(ψ) relates horizontal inertial velocities to body-fixed surge and sway velocities.

The internal PID loop regulates the body-fixed velocity error:

(10) $$e_{\nu }=\nu_{d}-\left[\begin{array}{c} u\\ v \end{array}\right],$$

producing the virtual force vector:

(11) $$\left[\begin{array}{c} X_{p}^{b}\\ Y_{p}^{b} \end{array}\right]=K_{p}^{\nu }e_{\nu }+K_{i}^{\nu }\int e_{\nu }dt+K_{d}^{\nu }{\dot{e}}_{\nu }.$$

Saturation blocks are inserted in both the integral action and the final control output to prevent windup and ensure boundedness of the commanded forces [26,27].

Considering the yaw control loop, the desired heading $\psi_{d}$ is computed from waypoint geometry using a LoS guidance law,

(12) $$\psi_{d}=\mathrm{atan}2\left(y_{d}^{I}-y^{I},\;x_{d}^{I}-x^{I}\right).$$

The yaw error is defined as

(13) $$e_{\psi }=\psi_{d}-\psi ,$$

and the cascade structure is similarly applied. The external proportional controller generates a desired yaw rate:

(14) $$r_{d}=k_{p}^{\psi }e_{\psi },$$

which feeds the internal PID controller:

(15) $$N_{p}^{b}=k_{p}^{r}\left(r_{d}-r\right)+k_{i}^{r}\int \left(r_{d}-r\right)dt+k_{d}^{r}\frac{d}{dt}\left(r_{d}-r\right).$$

The resulting $N_{p}^{b}$ corresponds to the desired yaw moment in the body-fixed frame.

Then the high- and low-level loops generate the Virtual Control Action (VCA) vector

(16) $$\tau_{p}^{b}=\left[\begin{array}{c} X_{p}^{b}\\ Y_{p}^{b}\\ N_{p}^{b} \end{array}\right],$$

which does not directly correspond to physical actuators.

Instead, due to the presence of six real control actuators (six propulsion motors) and only three VCAs, the system is overactuated [26,28].

It is also important to highlight that low-pass filters are applied to derivative terms to attenuate high-frequency noise and prevent amplification of measurement disturbances. The cutoff frequency of each filter is selected at least five times lower than the respective loop sampling frequency to preserve phase margin while attenuating undesirable oscillations [27].

Integral anti-windup mechanisms are implemented using conditional integration with saturation feedback, ensuring stability even under prolonged actuator saturation [29].

The resulting cascade P–PID structure provides:

- Decoupled position and velocity regulation;
- Improved disturbance rejection in surge–sway dynamics;
- Smooth yaw stabilization under aggressive maneuvering;
- Compatibility with nonlinear control allocation in overactuated conditions.

This hierarchical architecture preserves modularity between the guidance, control, and allocation layers, enabling independent tuning and ensuring real-time feasibility within the embedded Lua execution environment.

### Nonlinear Control Allocation Strategy

The propulsion architecture of the proposed vessel is based on classical control allocation theory, which formulates this problem as the minimization

(17) $$\min_{u_{v}}\;\;{∥W\left(u_{v}\right)u_{v}∥}_{2}\;\mathrm{subject}\;\mathrm{to}\;\tau_{p}^{b}=Mu_{v},$$

where τ denotes the generalized force vector, $u_{v}$ the actuator inputs, and M the control effectiveness matrix [30].

In many marine applications, this leads to pseudo-inverse or constrained quadratic programming solutions. However, such approaches may become computationally expensive or numerically ill-conditioned in embedded environments, particularly under frequent thrust reversals and actuator saturation [26,31].

In the HydroAir vessel, the high-level controller operates in the full planar subspace with three DoFs: surge, sway, and yaw [23]. Then, the VCA vector is therefore redefined as

(18) $$\tau_{p}^{b}=\left[\begin{array}{c} X_{p}^{b}\\ Y_{p}^{b}\\ N_{p}^{b} \end{array}\right]\in R^{3}.$$

Rather than directly solving $\tau_{p}^{b}=Mu_{v}$ through matrix inversion, the proposed allocation defines a nonlinear mapping

(19) $$\Phi :R^{3}\to R^{m},\;u=\Phi \left(u_{v}\right),$$

where m=6 is the Autonomous Surface Vehicle (ASV)’ actuator quantity, def here the actuator vector is defined as $u_{v}=\left[\delta_{1},\delta_{2},\delta_{3},\delta_{4},\delta_{5},\delta_{6}\right]$, constructed to preserve boundedness, homogeneity, and directional consistency in $R^{3}$.

First, the Euclidean magnitude of the VCAs is computed as

(20) $$\begin{array}{ccc} h & = & {∥\tau_{p}^{b}∥}_{2}+\varepsilon , \end{array}$$

(21) $$\begin{array}{ccc} & = & \sqrt{{X_{p}^{b}}^{2}+{Y_{p}^{b}}^{2}+{N_{p}^{b}}^{2}}+\varepsilon , \end{array}$$

with ε>0 ensuring differentiability at the origin. This normalization induces the mapping

(22) $${\tilde{\tau }}_{p}^{b}=\frac{\tau_{p}^{b}}{{∥\tau_{p}^{b}∥}_{2}+\varepsilon },$$

which projects the command vector onto the unit sphere in $R^{3}$. Consequently, outside the singular neighborhood controlled by ε, the mapping satisfies the positive homogeneity property

(23) $$\Phi \left(\lambda \tau_{p}^{b}\right)=\lambda \Phi \left(\tau_{p}^{b}\right),\;\forall \lambda \geq 0.$$

A maximum allocation gain α>0 defines the admissible generalized force envelope,

(24) $$n=\alpha {\tilde{\tau }}_{p}^{b}=\left[\begin{array}{c} n_{X}\\ n_{Y}\\ n_{N} \end{array}\right].$$

Instead of preserving the Euclidean structure of n, the allocation redistributes authority through a nonlinear $L_{1}$-based weighting extended to three dimensions:

(25) $$T_{w}=\frac{|n_{X}|}{|n_{X}\left|+\right|n_{Y}\left|+\right|n_{N}|+\varepsilon },\;L_{w}=\frac{|n_{Y}|}{|n_{X}\left|+\right|n_{Y}\left|+\right|n_{N}|+\varepsilon },\;S_{w}=\frac{|n_{N}|}{|n_{X}\left|+\right|n_{Y}\left|+\right|n_{N}|+\varepsilon }.$$

This defines a smooth mapping from the $L_{2}$-normalized sphere to the 3-simplex satisfying

(26) $$T_{w}+L_{w}+S_{w}=1-O\left(\varepsilon \right).$$

The effective force components are then defined as

(27) $$f_{X}=\alpha \;X_{p}^{b}\;T_{w},\;f_{Y}=\alpha \;Y_{p}^{b}\;L_{w},\;f_{N}=\alpha \;N_{p}^{b}\;S_{w}.$$

The generalized force vector after nonlinear redistribution becomes

(28) $$\tau_{e}=\left[\begin{array}{c} f_{X}\\ f_{Y}\\ f_{N} \end{array}\right].$$

The actuator mixing stage is defined through the full effectiveness matrix,

(29) $$u_{v}=M\tau_{e},$$

where $M\in R^{m\times 3}$ maps surge, sway, and yaw components into individual actuator commands. In the particular case of differential propulsion augmented with lateral actuation, M assumes the structured form

(30) $$M=\left[\begin{array}{ccc} 1 & 0 & 1\\ 1 & 0 & -1\\ 0 & 1 & 0 \end{array}\right].$$

Explicitly, the actuator commands become

(31) $$u_{R}=f_{X}+f_{N},\;u_{L}=f_{X}-f_{N},\;u_{Y}=f_{N}.$$

From a dynamical standpoint, substituting these expressions into the 3-DoF rigid-body model in Equation (2) preserves the standard marine vehicle structure while embedding a nonlinear bounded allocation layer prior to actuation in the full surge–sway–yaw space [23].

Actuator asymmetry is incorporated via the decomposition:

(32) $${u_{v}}_{i}^{+}=\max\left({u_{v}}_{i},0\right),\;{u_{v}}_{i}^{-}=\min\left({u_{v}}_{i},0\right),$$

which guarantees monotonic separation of forward and reverse thrust channels. The final PWM signal is generated by the affine transformation:

(33) $$PWM_{i}=PWM_{i,\mathrm{trim}}+k{u_{v}}_{i}.$$

It is important to emphasize that, unlike pseudo-inverse-based solutions,

(34) $$u_{v}=M^{†}\tau ,$$

which minimizes $∥u_{v}{∥}_{2}$ in a least-squares sense [30], the present formulation implements a nonlinear contraction mapping on the control input space in $R^{3}$, enforcing amplitude-bounded, direction-preserving allocation while explicitly incorporating the additional virtual control action associated with the third degree of freedom.

A more in-depth analysis of the proposed nonlinear allocation can be interpreted geometrically as a structured projection between normed spaces. Consider the VCA vector from Equation (18), defined in the generalized force space $R^{3}$. Then, the initial normalization step from Equation (22) corresponds to a radial projection onto the closed $L_{2}$ unit sphere:

(35) $$B_{2}=\left\{\left(X_{p}^{b},Y_{p}^{b},N_{p}^{b}\right)\in R^{3}:{X_{p}^{b}}^{2}+{Y_{p}^{b}}^{2}+{N_{p}^{b}}^{2}\leq 1\right\}.$$

However, actuator saturation in multi-actuator propulsion systems is more accurately represented in the actuator space $R^{6}$ by an $L_{1}$-type constraint of the form:

(36) $$U_{v}=\left\{u_{v}\in R^{6}:\sum_{i=1}^{6}\left|{u_{i}}_{v}\right|\leq {u_{v}}_{\max}\right\},$$

which defines an eight-dimensional cross-polytope describing the admissible actuator effort.

The corresponding admissible generalized force set is obtained as the linear image of $U_{v}$ through the configuration matrix $M\in R^{3\times 6}$,

(37) $$T=\left\{\tau \in R^{3}:\tau =Mu_{v},\;u_{v}\in U_{v}\right\}.$$

Geometrically, T is a three-dimensional zonotope whose shape depends explicitly on the actuator configuration matrix M. Therefore, while the normalization step operates in the spherical $L_{2}$ geometry of $R^{3}$, the saturation mechanism is intrinsically governed by the polyhedral structure induced by the $L_{1}$ constraint in $R^{8}$:

(38) $$\Phi :B_{2}\to B_{1},$$

which defines a continuous nonlinear mapping from the Euclidean unit sphere onto the actuator-feasible polytope in $R^{3}$.

Geometrically, the transformation can be interpreted as a norm-consistent reshaping between convex bounded sets:

- The $L_{2}$ normalization preserves directional information and ensures boundedness within the spherical envelope;
- The subsequent $L_{1}$ redistribution reshapes the admissible region into an octahedral geometry that better matches independent actuator saturation limits;
- The composition prevents multi-axis diagonal saturation, since the vertices of $B_{1}$ correspond to maximal single-axis thrust conditions.

**Figure 7:**
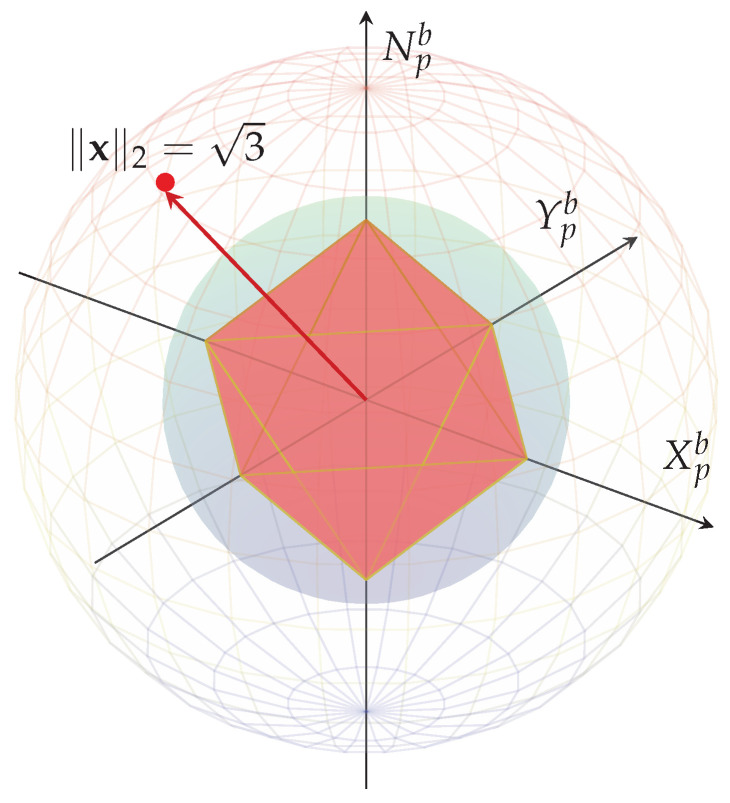
Geometric relationship between norm-bounded sets in $R^{3}$. The blue surface represents the Euclidean unit sphere $B_{2}$. The red octahedron corresponds to the $L_{1}$ unit sphere $B_{1}$. The faint outer sphere represents the scaled set $\sqrt{3}B_{2}$. The inclusions $B_{2}\subset B_{1}\subset \sqrt{3}B_{2}$ are visually highlighted.

Furthermore, the geometric relationship between both norm-bounded regions is illustrated in Figure 7.

In $R^{3}$, norm equivalence implies ${∥x∥}_{2}\leq {∥x∥}_{1}\leq \sqrt{3}\;{∥x∥}_{2}$, following that

(39) $$B_{2}\subset B_{1}\;\mathrm{and}\;B_{1}\subset \sqrt{3}\;B_{2}.$$

Hence every $L_{2}$-bounded command lies inside the $L_{1}$ unit sphere, while the $L_{1}$ unit sphere itself remains contained within a scaled Euclidean sphere. The nonlinear weighting, therefore, acts as a contraction toward the actuator-feasible polytope while preserving directional intent in the three-degree-of-freedom force space.

## 6. Experimental Results

This section presents the open-field experimental results obtained using the developed ASV prototype during field trials conducted in a dam environment. The experiments were designed to evaluate the performance of the proposed ASV and control architecture under realistic operating conditions, focusing on the vehicle navigation behavior in terms of inertial position, body-frame velocities, and heading dynamics.

The tests are divided into three subsections:

- Section 6.1 presents the first navigation scenario, in which the vessel performs a round-trip trajectory returning approximately to the starting point, allowing the evaluation of positioning consistency and navigation stability;
- Section 6.2 describes a maneuvering experiment composed of a figure-eight trajectory, which imposes distinct heading variations and allows assessing the dynamic behavior of the platform during continuous turning maneuvers;
- Section 6.3 illustrates a practical application of the HydroAir platform in an environmental reconstruction task, where the vehicle collects spatial data for generating bathymetric and aerial reconstructions of the reservoir environment.

Together, these experiments demonstrate the operational capability of the proposed system and highlight the effectiveness of the navigation and control strategy in real-world aquatic environments.

### 6.1. Scenario 1: Straight-Line Propulsion Test

This experiment evaluates the navigation performance of the developed ASV during a simple round-trip maneuver in open water. In this scenario, the vehicle starts from an initial position, moves along a straight trajectory, performs a turning maneuver, and returns approximately to the starting point. This experiment allows evaluating the stability of the closed-loop control system and the accumulated positioning error caused by GPS uncertainty and environmental disturbances.

**Figure 8:**
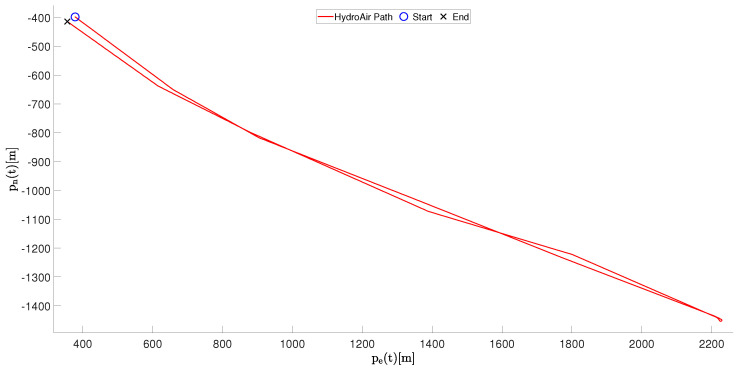
Inertial trajectory of the HydroAir vessel in the $\left(p_{north},p_{east}\right)$ plane during the straight-line navigation experiment in Scenario 1.

Then, Figure 8 illustrates the inertial position trajectory obtained during the experiment, represented by the North-East coordinates.

The trajectory shows the outbound motion followed by the return segment toward the initial position. As expected for GNSS measurements operating in open environments, the final position does not perfectly coincide with the starting point. The resulting positioning discrepancy is mainly due to GPS noise, slow vessel dynamics, and small disturbances from water currents and wind.

Quantitatively, the total traveled distance during the experiment was approximately 2.1 km, with a mean forward velocity of approximately 1.2852 m/s during the outbound motion and a peak surge velocity of 1.4330 m/s. During the turning maneuver, the yaw rate reached approximately 30 deg/s.

**Figure 9:**
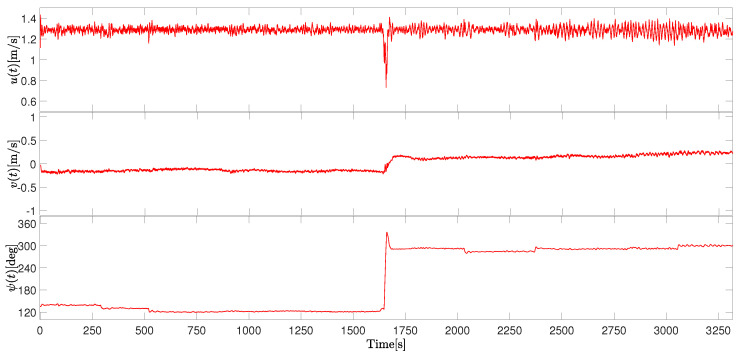
Time evolution of the vessel linear velocities (including surge velocity *u*, sway velocity *v*) and the vessel angular position (yaw angle ψ) in Scenario 1.

For the presented results, Figure 9 presents the body-frame velocity components.

The surge velocity *u* remained predominantly positive during the outbound trajectory and decreased as the vessel approached the turning region. The sway velocity *v* remained close to zero during straight motion and increased slightly during the turning maneuver, reaching values around 0.2861 m/s. This behavior is expected for marine vehicles performing heading adjustments facing water current and wind interference. For the yaw angle ψ, the heading angle varies smoothly during the whole straight part, performing hard maneuvers only at the turning point, 1625 s. The maximum heading change during the maneuver was approximately 171 degrees.

Overall, the experiment demonstrates that the developed ASV and its control techniques were capable of producing stable motion and consistent trajectory tracking, even during straight-line operation, in a real-world environment.

### 6.2. Scenario 2: Heading Maneuver and Turning Motion

The second experiment evaluates the performance of the developed ASV under different maneuvering conditions. In this scenario, the vehicle performs multiple trajectory segments forming a figure-eight pattern. This test allows assessing the control system behavior during continuous heading changes, as well as the robustness of the propulsion and steering allocation strategy.

**Figure 10:**
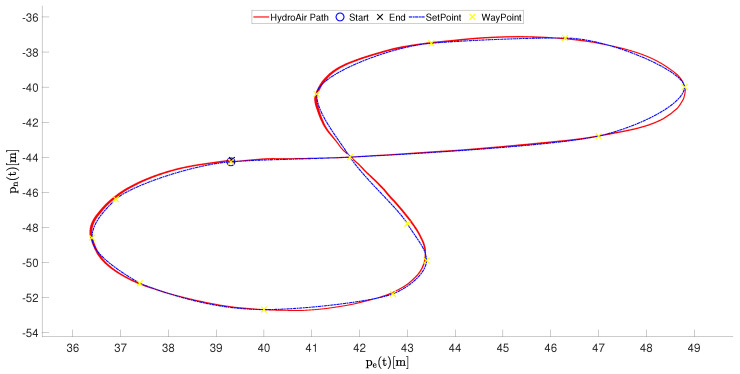
Inertial trajectory of the HydroAir vessel in the $\left(p_{north},p_{east}\right)$ plane during the eight-pattern navigation experiment in Scenario 2.

Then, Figure 10 shows the inertial trajectory of the vessel in the North-East plane.

The resulting path clearly forms a figure-eight shape, requiring alternating left- and right-hand turns. This configuration represents a more demanding navigation task than the first scenario, since the controller must continuously regulate both translational motion and the heading angle.

The total trajectory length during the experiment was approximately 66 m, distributed over 1 loop. The average forward velocity was approximately 1.0269 m/s, while the peak velocity reached 1.4871 m/s during the straight segments of the trajectory. Despite continuous maneuvering, the vessel maintained a smooth, stable motion, with no noticeable oscillations or trajectory divergence. As a consequence, these signals exhibit alternating increases and decreases in the East coordinate while the North coordinate evolves more gradually. The smooth variation of these signals indicates consistent navigation performance across successive maneuvers.

**Figure 11:**
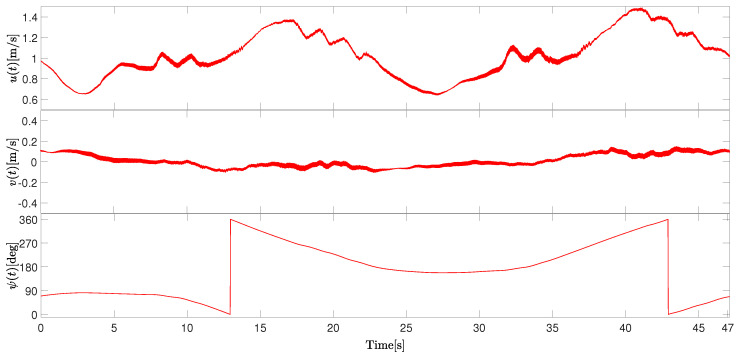
Time evolution of the vessel linear velocities (including surge velocity *u*, sway velocity *v*), and the vessel angular position (yaw angle ψ), in Scenario 2.

The body-frame velocity components are presented in Figure 11:

The sway velocity remains relatively stable throughout the experiment, oscillating around an average value of approximately 0.1 m/s. In contrast, the sway velocity exhibits periodic variations associated with the alternating turning maneuvers, as expected. The peak sway velocity observed during the experiment was smooth, almost disregarded. Furthermore, the heading angle ψ presented a periodic pattern as the vessel alternated between left and right turns while tracing the figure-eight trajectory. The total heading variation during each loop is approximately one complete loop, i.e., 360 degrees.

Again, the results demonstrated that the proposed vehicle is capable of performing distinct maneuvering tasks (independent of direction) while maintaining stable velocity. The experiments also confirm the suitability of the HydroAir platform for low-speed autonomous navigation in open-water environments.

### 6.3. Environmental Reconstruction Demonstration Using the HydroAir Platform

Although the primary focus of this work is the whole ASV, the developed HydroAir platform was designed for environmental sensing applications through the integration of onboard sensors and aerial data acquisition.

To demonstrate the system’s operational capability and stability under real conditions, a field experiment was conducted in a reservoir environment, where the platform was used to perform a preliminary reconstruction of the surrounding terrain and underwater structures.

During the experiment, the ASV navigated through the test area while carrying a depth-sensing device capable of measuring the vertical distance between the water surface and the reservoir bed. Simultaneously, aerial images were collected using an UAV, allowing the reconstruction of the surrounding environment above the waterline. The combination of these sensing modalities enables the generation of a hybrid environmental representation that includes both aerial terrain and underwater bathymetry.

**Figure 12:**
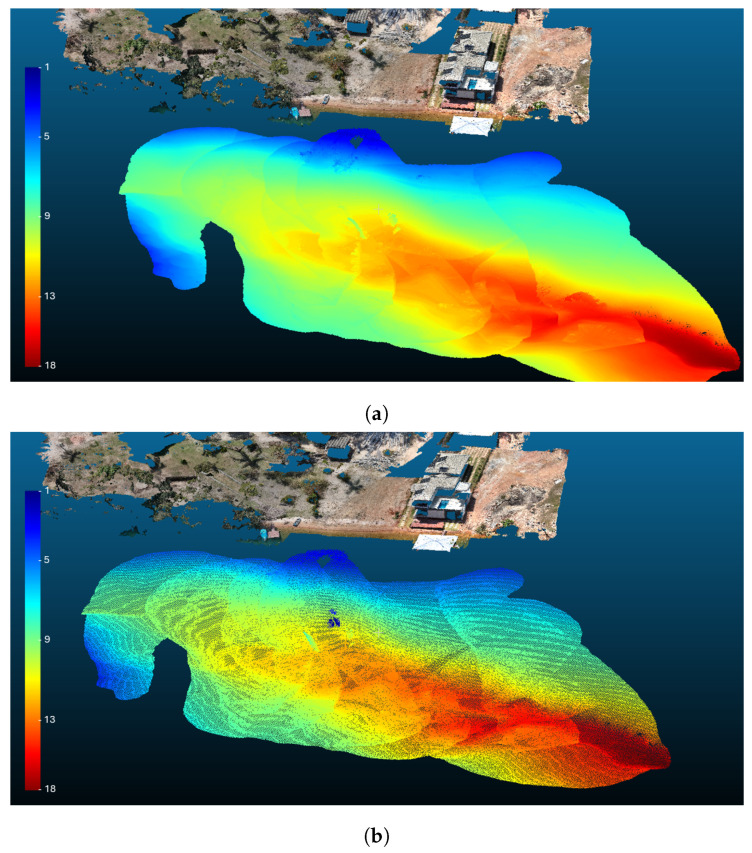
Environmental reconstruction generated using data collected by the HydroAir platform during field experiments. (**a**) Surface mesh reconstruction of the surveyed area combining aerial and underwater data. (**b**) Point cloud representation of the reconstructed environment.

The resulting reconstruction is illustrated in Figure 12.

The images present a three-dimensional representation of the surveyed area, where the upper region corresponds to the reconstructed terrain derived from aerial imagery, and the lower region corresponds to the underwater surface generated from depth measurements collected by the HydroAir platform during navigation.

Color intensity in the reconstructed underwater region represents the relative depth of the reservoir bed. Warmer colors, such as red and orange, indicate deeper regions of the reservoir, while cooler colors, such as green and blue, represent shallower areas. This representation allows a quick visual interpretation of the bathymetric profile of the surveyed environment.

It is important to emphasize that the environmental reconstruction itself is not the central objective of this work. Instead, this experiment serves as a practical validation scenario that illustrates the HydroAir platform’s ability to operate autonomously while transporting sensing payloads and collecting environmental data in real-world conditions. The vehicle’s stability, ensured by the proposed control framework and control allocation strategy, is a key requirement, previously shown.

The results demonstrate that the HydroAir allows for coordinated data acquisition in aquatic environments, supporting applications such as environmental monitoring, bathymetric mapping, and infrastructure inspection in reservoirs and other inland waters.

## 7. Conclusions

This paper presents the development, control architecture, and experimental validation of the HydroAir ASV prototype, designed for autonomous navigation and environmental monitoring tasks in aquatic environments, including shallow water. The proposed system integrates a modular control architecture with a nonlinear control allocation strategy, enabling the coordinated generation of surge, sway, and yaw actions required for stable planar motion.

The experimental results from open-field trials demonstrated the platform’s operational capability under realistic conditions. In the first navigation scenario, the vessel followed a straight-line round-trip trajectory, returning approximately to its initial location, with an average surge velocity of 1.2852 m/s. Given the inherent limitations of GNSS measurements and the vessel’s low speed, this level of positioning accuracy is acceptable for autonomous surface navigation in reservoir environments.

The second experimental scenario evaluated the system under maneuvering conditions through a figure-eight trajectory. The results show smooth, continuous evolution of the inertial position and velocity signals, with average surge velocities of 1.0269 m/s. The navigation signals remained bounded and stable throughout the experiment, indicating that the proposed control strategy can handle continuous heading changes without introducing oscillatory behavior.

Additionally, the experiments confirmed that the HydroAir platform can operate reliably in real-world conditions, maintaining stable motion while executing different navigation patterns. The obtained trajectories and velocity profiles demonstrate that the implemented control architecture provides adequate performance for low-speed autonomous surface vehicles operating in inland water bodies.

Future work will focus on extending the experimental validation to more complex missions involving waypoint tracking and autonomous mission planning. Additional improvements will include integrating enhanced state estimation methods, such as sensor fusion between GNSS and inertial measurements, and evaluating the platform under stronger environmental disturbances, such as strong winds and water currents.

## Figures and Tables

**Table 1:** HydroAir inertial parameters.

Parameter	Value	Unit
*m*	192.9	kg
$I_{z}$	130.3	kg·m^2^
$x_{g}$	−1.33	[m]
$y_{g}$	0.00	[m]

**Table 2:** Summary of the parameter estimates. Non-mentioned parameters are considered null.

Parameter	Value	Unit
$X_{\dot{u}}$	−16.26	kg
$Y_{\dot{v}}$	−122.8	kg
$N_{\dot{r}}$	−40.60	kg·m^2^
$X_{|u|u}$	−13.17	kg/m
$Y_{|v|v}$	−576.5	kg/m
$N_{|r|r}$	−38.22	kg·m^2^/rad^2^

## Data Availability

The data used to support the findings of this study are available from the corresponding author upon request.

## References

[1] Liu Z., Zhang Y., Yu X., Yuan C. (2016). Unmanned surface vehicles: An overview of developments and challenges. Annu. Rev. Control.

[2] Bae I., Hong J. (2023). Survey on the Developments of Unmanned Marine Vehicles: Intelligence and Cooperation. Sensors.

[3] Fascista A. (2022). Toward Integrated Large-Scale Environmental Monitoring Using WSN/UAV/Crowdsensing: A Review of Applications, Signal Processing, and Future Perspectives. Sensors.

[4] Tang K.K.W., Mohd Yatim M.H., Darwin N., Wan Aris W.A., Yen S.C., Mohamed Fadil N. (2025). Seamless Multisource Topo-Bathymetric Elevation Modelling for River Basins: A Review of UAV and USV Integration Techniques. Rev. Int. Geomat..

[5] Patterson R.G., Cronin M.F., Swart S., Beja J., Edholm J.M., McKenna J., Palter J.B., Parker A., Addey C.I., Boone W. (2025). Uncrewed surface vehicles in the Global Ocean Observing System: A new frontier for observing and monitoring at the air-sea interface. Front. Mar. Sci..

[6] Chu Y., Gao Q., Yue Y., Lim E.G., Paoletti P., Ma J., Zhu X. (2024). Evolution of Unmanned Surface Vehicle Path Planning: A Comprehensive Review of Basic, Responsive, and Advanced Strategic Pathfinders. Drones.

[7] Kovanič Ľ., Topitzer B., Peťovský P., Blišťan P., Gergeľová M.B., Blišťanová M. (2023). Review of Photogrammetric and Lidar Applications of UAV. Appl. Sci..

[8] Mohsan S., Khan M.A., Noor F., Ullah I., Alsharif M.H. (2022). Towards the Unmanned Aerial Vehicles (UAVs): A Comprehensive Review. Drones.

[9] Mohsan S., Othman N.Q.H., Li Y., Alsharif M.H., Khan M.A. (2023). Unmanned aerial vehicles (UAVs): Practical aspects, applications, open challenges, security issues, and future trends. Intell. Serv. Robot..

[10] Wibisono A., Piran M.J., Shin H.K., Lee B.M. (2023). A Survey on Unmanned Underwater Vehicles: Challenges, Enabling Technologies, and Future Research Directions. Sensors.

[11] Liu X., Ho L.T., Bruneel S., Goethals P.L.M. (2025). Applications of unmanned vehicle systems for multi-spatial scale monitoring and management of aquatic ecosystems: A review. Ecol. Inform..

[12] Xing B., Yu M., Liu Z., Tan Y., Sun Y., Li B. (2023). A Review of Path Planning for Unmanned Surface Vehicles. J. Mar. Sci. Eng..

[13] Hashali S.D., Yang S., Xiang X. (2024). Route Planning Algorithms for Unmanned Surface Vehicles (USVs): A Comprehensive Analysis. J. Mar. Sci. Eng..

[14] Wu Y., Wang T., Liu S. (2024). A Review of Path Planning Methods for Marine Autonomous Surface Vehicles. J. Mar. Sci. Eng..

[15] Vagale A., Oucheikh R., Bye R.T., Osen O.L., Fossen T.I. (2021). Path planning and collision avoidance for autonomous surface vehicles I: A review. J. Mar. Sci. Technol..

[16] de Andrade E.M., Sales J.S., Fernandes A.C. (2025). Operative Unmanned Surface Vessels (USVs): A Review of Market-Ready Solutions. Automation.

[17] Zhang G., Valero D., Bung D.B., Chanson H. (2018). On the estimation of free-surface turbulence using ultrasonic sensors. Flow Meas. Instrum..

[18] Alharbi O., Kane T., Henderson D. (2022). Impact of a Turbulent Ocean Surface on Laser Beam Propagation. Sensors.

[19] Tang X., Pei Z., Yin S., Li C., Wang P., Wang Y., Wu Z. (2020). Practical design and implementation of an autonomous surface vessel prototype: Navigation and control. Int. J. Adv. Robot. Syst..

[20] Wang W., Gheneti B., Mateos L.A., Duarte F., Ratti C., Rus D. (2019). Roboat: An autonomous surface vehicle for urban waterways. 2019 IEEE/RSJ International Conference on Intelligent Robots and Systems (IROS).

[21] Rynne P.F., von Ellenrieder K.D. (2010). Development and preliminary experimental validation of a wind- and solar-powered autonomous surface vehicle. IEEE J. Ocean. Eng..

[22] Fossen T.I. (1994). Guidance and Control of Ocean Vehicles.

[23] Fossen T.I. (2011). Handbook of Marine Craft Hydrodynamics and Motion Control.

[24] dos Santos Neto A.F., de Mello Honório L., da Silva M.F., da Silva Júnuir I.C., Westin L.G.F. (2021). Development of Optimal Parameter Estimation Methodologies Applied to a 3 DOF Autonomous Surface Vessel. IEEE Access.

[25] Dos Santos M.F., Neto A.F.D.S., Honório L.D.M., Da Silva M.F., Mercorelli P. (2023). Robust and optimal control designed for autonomous surface vessel prototypes. IEEE Access.

[26] Santos M., Honório L., Moreira A.P.G.M., Garcia P.A.N., Silva M., Vidal V.F. (2022). Analysis of a fast control allocation approach for nonlinear over-actuated systems. ISA Trans..

[27] Leal Lopes V.M., Honório L.M., Santos M.F., Pancoti A.A., Silva M.F., Diniz L.F., Mercorelli P. (2023). Design of an Over-Actuated Hexacopter Tilt-Rotor for Landing and Coupling in Power Transmission Lines. Drones.

[28] da Silva M.F., Honório L.M., Marcato A.L.M., Vidal V.F., Santos M.F. (2020). Unmanned aerial vehicle for transmission line inspection using an extended Kalman filter with colored electromagnetic interference. ISA Trans..

[29] Silva M.F., Ribeiro A.C., Santos M.F., Carmo M.J., Honório L.M., Oliveira E.J., Vidal V.F. Design of angular PID controllers for quadcopters built with low cost equipment. Proceedings of the 20th International Conference on System Theory, Control and Computing (ICSTCC).

[30] Johansen T.A., Fossen T.I. (2013). Control allocation—A survey. Automatica.

[31] dos Santos M.F., de Mello Honório L., Moreira A.P.G.M., da Silva M.F., Vidal V.F. (2021). Fast Real-Time Control Allocation Applied to Over-Actuated Quadrotor Tilt-Rotor. J. Intell. Robot. Syst..

